# Preliminary Examination of the Toxicity of Spalting Fungal Pigments: A Comparison between Extraction Methods

**DOI:** 10.3390/jof7020155

**Published:** 2021-02-22

**Authors:** Badria H. Almurshidi, R.C. Van Court, Sarath M. Vega Gutierrez, Stacey Harper, Bryan Harper, Seri C. Robinson

**Affiliations:** 1 Department of Wood Science, Oregon State University, Corvallis, OR 97333, USA; badria@email.sc.edu (B.H.A.); ray.vancourt@oregonstate.edu (R.C.V.C.); sarathth@yahoo.co.uk (S.M.V.G.); 2 Department of Toxicology, Oregon State University, Corvallis, OR 97331, USA; Stacey.Harper@oregonstate.edu (S.H.); Bryan.Harper@oregonstate.edu (B.H.)

**Keywords:** spalting, fungal pigment, xylindein, dramada, *Chlorociboria aeruginosa*, *Chlorociboria aeruginascens*, *Scytalidium cuboideum*, natural pigment, natural colorant

## Abstract

Spalting fungal pigments have shown potential in technologies ranging from green energy generation to natural colorants. However, their unknown toxicity has been a barrier to industrial adoption. In order to gain an understanding of the safety of the pigments, zebrafish embryos were exposed to multiple forms of liquid media and solvent-extracted pigments with concentrations of purified pigment ranging from 0 to 50 mM from *Chlorociboria aeruginosa, Chlorociboria aeruginascens,* and *Scytalidium cuboideum.* Purified xylindein from *Chlorociboria sp*. did not show toxicity at any tested concentration, while the red pigment dramada from *S. cuboideum* was only associated with significant toxicity above 23.2 uM. However, liquid cultures and pigment extracted into dichloromethane (DCM) showed toxicity, suggesting the co-production of bioactive secondary metabolites. Future research on purification and the bioavailability of the red dramada pigment will be important to identify appropriate use; however, purified forms of the blue-green pigment xylindein are likely safe for use across industries. This opens the door to the adoption of green technologies based on these pigments, with potential to replace synthetic colorants and less stable natural pigments.

## 1. Introduction

Spalting fungi are a specific group of wood decay fungi that have the ability to internally color wood [1]. The coloration that they cause can be classified into three types: bleaching, zone lines, and pigmentation. The first two types are produced mostly by white-rotting fungi. Pigmentation is caused by ascomycete fungi through the generation of secondary metabolites that cause coloration in wood. Known colors produced by these fungi include blue-green produced by *Chlorociboria* spp. [2,3,4], red from *Scytalidium cuboideum* (Sacc. and Ellis) Sigler and Kang [5], and yellow from *Scytalidium ganodermophthorum* Sigler and Kang [6], among others.

**Figure 1:**
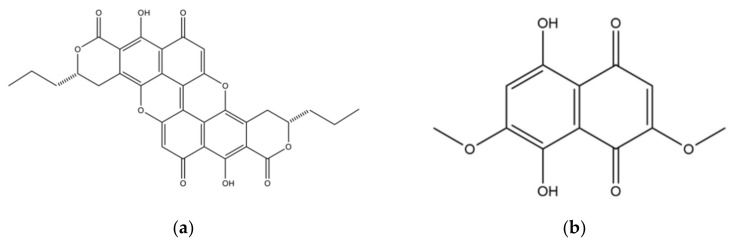
Structure of spalting fungal pigments. (**a**) Blue/green pigment xylindein produced by *Chlorociboria spp.*; (**b**) red pigment “dramada” (5,8-Dihydroxy-2,7-dimethoxy-1,4-naphthoquinone) produced by *Scytalidium cuboideum.*

Beginning in the 15th century, wood stained blue-green by fungi from the genus *Chlorociboria* was a prized commodity in fine woodworking [7,8,9]. Artworks containing the blue-green pigment, named xylindein [10] (Figure 1), retain their coloration today, attesting to its stability. The structure of this pigment has since been established [11,12,13], and its impressive UV stability and thermal stability have been the subject of research [14]. The properties of other pigments have also been the subject of recent investigation, including the identification of the napthoquinonic crystal produced by *Scytalidium cuboideum,* called dramada [15]. This red compound has also been isolated from an actinomycete [16].

Pigments produced by these fungi have been investigated for their use in a variety of fields, for example, as a coloring agent for wood stain [17], in paint [18], and as a textile dye [19,20,21]. The use of fungal pigments in these applications could replace current unsustainable industrial practices. For example, conventional textile dyeing practices produce toxic wastewater associated with negative environmental and health consequences [22,23,24,25]. The use of sustainably produced fungal pigments instead would allow for the production of desired colored cloth without the toxic tradeoff. In addition to use as a colorant, the pigments have also been the subject of investigation into use as organic semiconductors [14,26], and may allow for fully sustainable energy generation through organic photovoltaic systems. However, before the adoption of these green technologies is possible, especially for those associated with extended human contact, the toxicity of the pigments must be understood. 

Many filamentous fungi produce secondary metabolites with bioactive effects. Some have a beneficial effect, with pharmacological uses like antibiotics [27,28]. However, fungi also produce a range of mycotoxins such as aflatoxins and rhizonin, which are severe health hazards [29]. A number of filamentous fungi also produce pigments that are themselves toxic [30]. For example, pigments from *Monascus* spp., used for coloring food and in pharmacological applications, are restricted due to fungal co-production of citrinin [31], which is nephrotic [32]. This has led to research attempting to reduce citrinin production through growth condition variation or strains [33,34,35,36,37].

Spalting fungi have received attention for their potential associated health risks, most notably by woodturners who have spread fear about their supposed toxicity. Theories spread have included spalted wood causing allergic reactions, releasing “carloads” of spores, and implications of brain infection, which have likely been driven through limited understanding of fungal biology [38]. These and more urban myths around any potential threats that spalted wood might pose compared to non-decayed wood have been debunked, as spalted wood is not inherently more dangerous or toxic than non-decayed wood [39]. 

To determine the potential hazard of toxins in humans, a broadly used method involves the testing of compounds in zebrafish (*Danio rerio*) embryos. Zebrafish are a tropical freshwater fish that have been highly studied and used as a model organism for rapid and low-cost research in the fields of toxicology, genetics and developmental biology [40,41,42,43]. Zebrafish assays are used to indicate bioactive drugs and therapeutic compounds for pharmaceutical applications and to understand effects on developmental mechanisms [44,45,46,47]. The use of the zebrafish embryo model for toxicology research is accepted internationally as an alternate animal model on testing hazard and risk assessment [48,49]. The zebrafish model has also been used to evaluate the toxicity of natural products from plants and other organisms such as secondary metabolite extracts [50,51,52], making it an appropriate model for preliminary examination of the toxicity of spalting fungal pigments.

This study sought to characterize the potential toxic effects of spalting fungal pigments and identify whether they came from the pigments themselves or due to the presence of other compounds in the extract. Understanding the toxicity of these pigments, as well as the cause (whether the pigment *itself* is toxic or the accompanying secondary metabolites are toxic), will enable the determination of what products they are appropriate for use in, and what level of processing after fungal production they require to be safely used. This information will inform future research and industrial adoption of these unique, sustainably sourced pigments.

## 2. Materials and Methods

### 2.1. Preparation of Pigment Samples

#### 2.1.1. Solvent-Extracted Pigment

*Chlorociboria aeruginosa* UAMH 11657 (isolated from a decaying hardwood log in Haliburton, ON, Canada), *Chlorociboria aeruginascens* UAMH 7615 (isolated in Lake District, UK), and Scytalidium *cuboideum* (Sacc. and Ellis) Sigler and Kang UAMH 11517 (isolated from *Quercus* sp. in Memphis, TN, USA) were used to inoculate petri dishes containing 2% malt extract agar (MEA) (20 g of bacteriological malt extract (VWR, Radnor, PA, USA), 15 g of agar (VWR), 1 l of deionized water) amended with sterile white rotted wood chips from either *Acer saccharum* or *Populus grandidentata*, following the protocol set by Robinson et al. [53]. Cultures were harvested once plates were completely pigmented, with times ranging from four (*Scytalidium cuboideum*) to twelve weeks (*Chlorociboria* spp.). Plates were opened and left to dry for 48 h, then ground using a blender (Oster Precise Blend, Boca Raton, FL, USA) until reaching a maximum size of ~5 mm. The resulting powder and 45 mL of dichloromethane (DCM) (VWR, Radnor, PA, USA) were combined in a 250 mL Erlenmeyer flask with a 2 mm × 5 mm VWR Spinbar magnetic stir bar. The flask was closed with a rubber cap and a stirred at 220 rpm for 30 min on a VWR Dylastir stir plate. The resulting solution was then filtered through VWR 415 Whatman Filter Paper to remove the wood chip particles. The extract was collected in a borosilicate glass vial (Ace glass, Vineland, NJ, USA) and sealed with non-evaporative polyseal-cone-lined caps. 

#### 2.1.2. Pigments from Liquid Media 

Liquid media were prepared following methods in Weber et al. [54]. Sterilized and cooled 150 mL mason jars containing 50 mL of 2% malt broth (20 g of VWR bacteriological malt extract, 1 l of deionized water) were inoculated with active fungal cultures of either *C. aeruginosa* or *S. cuboideum* using one plug of approximately 2 mm in diameter. Jars were then incubated at room temperature (21 °C) for 28 days on an open shelf. 

Pigment from liquid media was tested in three ways. First, liquid media were used directly in zebrafish assays. Second, liquid media were autoclaved at 121 °C for 30 min. Finally, media from fungal liquid malt cultures were also cleaned independently using Strata SPE 2 g/12 mL columns (Phenomenex). The column was conditioned by adding 4 mL of HPLC acetonitrile (CAN) solvent to remove trapped air and activate the SPE particles, before the solvent was removed and 4 mL HPLC grade water was added to maximize the sorbent interaction with target analytes. For all species, liquid media culture was filtered through 415 Whatman filter paper (VWR) twice before 10 mL was loaded onto activated column, where a visible band of pigment was formed. Contaminants were removed from the column through the addition of 10 mL 50% acetonitrile (ACN) in HPLC grade water. Pigment was eluted using 2 to 4 mL of 100% of HPLC-grade chloroform (EMD Millipore, Burlington, MA, USA). About 10 mL of the pigment mixture sample was used to achieve less than 0.5 mL of each purified pigment in two to three hours. This method was used to obtain pigment with a reduced amount of contaminants to reduce and identify potential effects of the extracts on the zebrafish embryos. 

#### 2.1.3. Solid Pigments

*Scytalidium cuboideum* pigment (dramada) crystals were precipitated by applying 200 mL of liquid nitrogen to a solution of 100 mL of concentrated acetone extract from *S. cuboideum* following the method stated by [15]. The acetone carrier differed from the traditional DCM as its melting point (−95 ° C) was preferred for the crystallization by precipitation method. After the crystals were formed in the cold solvent, they were filtered with the use of 415 Whatman filter paper (VWR). The crystals were then air-dried and placed in a glass vial. This method has been shown to result in crystals of high purity [15,55], with samples tested for purity in previous work [55].

To obtain solid xylindein from *Chlorociboria* spp., 15 mL of standardized *Chlorociboria* spp. extract in DCM was placed in a 30 mL ACE borosilicate glass vial and left uncovered to fully evaporate. Then, another 15 mL of extract was added. This process was repeated 15 times to form a solid pigment layer attached to the glass of the vial. Once the last fill of DCM extract was completely evaporated, 10 mL of acetone was added to the vial and it was closed with a non-evaporative polyseal-cone-lined cap. The vial was then shaken for one minute by hand before the mix was filtered using a VWR glass funnel equipped with a 415 Whatman filter paper to collect solid pigment. The acetone wash was repeated until the solid pigment was completely removed from the glass vial. After finishing this process, the filter paper was left to dry overnight. The resulting solid pigment was removed from the filter paper with the use of forceps and stored in a borosilicate glass vial. Solidified xylindein collected via this method does not yield a pure compound [56]; however, a standardized purification methodology has not yet been developed, though methods are in development [57].

#### 2.1.4. Solvent-Extracted Pigment Concentration

Standards corresponding to the extracts with DCM have been previously used to determine pigment concentration based on CIE L*a*b* values, not dry weight, when working with pigmenting spalting fungi [17,53,55,58]. The color values were used instead of the dry weight due to the simplicity of a direct extraction from the dry plates with DCM. The standard CIEL*a*b values utilized were established by [59] with a range of +/− 2.0 for each fungal species, including: *C. aeruginosa* L* = 82.28, a* = − 11.06, b* = −5.40 and *S. cuboideum* L* = 82.32, a* = 26.84, b* = 13.19. Three milliliters of each of the extracted solutions was added to a VWR glass cuvette for analysis on a Konica Minolta Chroma Meter CR−5 colorimeter, and concentration adjusted through the addition of solvent or evaporation to match these color values. After the read, the dye solution was returned to the vial and stored for future use.

### 2.2. Zebrafish Preparation and Exposure for Pigment Extracts

All experiments were performed in compliance with national care and use guidelines, and were approved by the Institutional Animal Care and Use Committee (IACUC) at Oregon State University (ACUP 5113). Adult wild-type D5 zebrafish (*Danio rerio*) embryos were raised at the Sinnhuber Aquatic Research Laboratory (SARL) at Oregon State University (Corvallis, Oregon, USA). Fish were maintained in fish water, consisting of reverse osmosis water supplemented with 0.3 g/l Instant Ocean salts (Aquatic Ecosystems, Apopka, FL) with pH adjusted with sodium bicarbonate to pH 7 ± 0.2, with a temperature of 28 ° C and a 14 h light to 10 h dark photoperiod. After group spawn and egg collection, an Olympus-SZ51 stereomicroscope was used to select and remove the abnormal and non-fertilized eggs. Six hours post-fertilization (hpf), all normal embryos were dechorionated to ensure contact with test materials [60]. Embryos were placed into a 60 mm glass petri dish with 25 mL fish water and exposed to 50 μL of 50 mg/mL pronase enzyme (Sigma-Aldrich, cat # 81750, St. Louis, MO, USA) to degrade the outer chorionic layer. After chorion deflation (~7 min) solution was diluted with fresh fish water and recovered in a petri dish at room temperature until 8 hpf, when waterborne exposure testing was carried out. At this time, each embryo was placed in its own well in a prepared 96-well plate containing fish water and tested pigment condition. The embryos were incubated at 28 °C for 24 hfp for the first assessment.

#### 2.2.1. Pigment from Solvent Extraction

The pigment bioactivity testing procedure using zebrafish embryos followed methods laid out in Truong, Harper and Tanguay [60]. Standardized pigments in DCM were placed in Zinser 96-well glass petri dishes and the solvent was allowed to evaporate under a fume hood for 24 h or until the DCM had evaporated completely, with 100 µL of each pigment extract used per well. At 8 hpf, 200 μL of fish water containing 0.3 g/L of aquatic salt was transferred with a VWR disposable wide-bore glass pipette in the Zinser 96-well glass petri dish, and one dechorionated embryo was added per well. These were then incubated at 28 °C until 24 hpf, then the appropriate assessments were performed as described below.

Extracted pigment toxicity was compared across multiple conditions for pigments from all tested fungal species. First, embryos were exposed to standardized pigments from fungi grown on aspen wood chip amended malt agar plates in one 96-well plate at 100% concentration (*n* = 12 for *Chlorociboria* species and *n* = 24 for *Scytalidium cuboideum*). Next, extracted pigment from fungi grown in maple amended wood chip plates at standard concentration was carried out in individual plates (*n* = 72 per pigment). Testing across concentrations was then carried out using only pigment extracts from amended maple wood chip plates. Each pigment extract was tested in separate 96-well plates using 72 embryos (*n* = 72 per pigment), with separate tests for 100%, 200%, and 400% concentrations. This range of concentrations was used to allow for comparison with previous publications using standardized pigment extract.

#### 2.2.2. Pigment from Liquid Culture

Three liquid culture solutions (live, autoclaved, and filtered, as described above) from each fungal species and SPE column purified pigment from *Chlorociboria* spp. and *S. cuboideum* were tested. These forms were tested in order to compare the toxicity of the extracted pigment solution to the full panel of compounds present in fungal cultures used to produce the pigments. For each, 100 μL of pigment solution was transferred into a Falcon sterile 96-well plate and fish water was added to give a final working volume of 250 μL, with controls of fresh fish water and sterile liquid malt extract. At 8 hpf, a dechorionated embryo was transferred into each individual well of the 96-well plate using a VWR disposable wide-bore glass pipette. The plates were incubated at 28 °C until 24 hpf, then assessed as described below.

First, pigmented liquid media taken directly from fungal cultures were applied to 12 embryos per pigment. This was then repeated using media autoclaved at 120 °C for 30 min. Next, pigment solutions were filtered to remove fungal cells from the medium using 0.2 µm EMD Millipore filters. This experiment was run twice, with 24 embryos per test condition each time. Finally, green pigment from *C. aeruginosa* and *C. aeruginascens* and red pigment from *S. cuboideum* collected from liquid media samples and purified using SPE columns (as described in Section 2.1.2) were tested on 72 embryos each. 

#### 2.2.3. Assessment Protocol

The assessment method was modified from Truong, Harper and Tanguay [60]. At 24 hpf, developmental stages and spontaneous kinetics were observed over a two-minute period. At 120 hpf, embryo morphology, including body axis, ocular perceiver, snout, jaw, notochord, heart, brain, somite, fin, yolk sac, trunk, circulation, pigment, swim bladder and behavioral endpoints (motility, tactile replication), was observed and recorded. Mortality rate was also recorded at 24 and 120 hpf. The assessments were conducted in a binary form, as present or not present.

### 2.3. Zebrafish Preparation and Exposure for Solid Purified Pigments

#### 2.3.1. Preparation of Zebrafish

Adult tropical 5D strain zebrafish (*Danio rerio*) embryos were collected and staged. Chorion was enzymatically removed using pronase (63.3 mg/mL) using a custom automated dechorionator [61] at 4 h past fertilization. 

#### 2.3.2. Exposure Protocol

Six concentrations of solidified xylindein from *C. aeruginosa* and dramada from *S. cuboideum* (as described in Section 2.1.3) were compared to aniline (≥99.5% ACS grade, Sigma-Aldrich). Tested concentrations ranged from 50 to 2.32 mM for dramada and aniline. Roughly the same concentrations were used for xylindein; however, as yet no method exists that results in a pure compound, so tested concentrations ranged from 28.42 mg/mL (~50 mM) to 1.27 mg/mL (~2.32 mM). Compounds were exposed in 96-well plates, with one embryo per well loaded at 6 hpf and 100 ul of exposure solution. There were a total of 32 embryos per concentration across multiple plates, with 8 embryos exposed per concentration per plate.

#### 2.3.3. Embryo Photomotor Response (EPR) Behavior

Embryos were assessed at 24 hpf using a custom photomotor response analysis tool [62] in plate. The light cycle consisted of 30 s of dark background, a short light pulse, followed by a second light pulse nine seconds later and 10 more seconds of dark. Every exposure plate had 850 frames of digital video recorded from below at 17 frames per second, with white LED and infrared lights above. Recorded periods were truncated at the beginning and end of the experiment to ensure the same recorded period was compared.

#### 2.3.4. Larval Photomotor Response Behavior (LPR)

Embryos were assessed at 120 hpf using a Zebrabox behavior chamber (ViewPoint Life Sciences, Montreal, CA, USA) with an infrared backlight stage. Total movement in response to three light cycles (3 min of light to 3 min dark) was tracked in 96 wells during a 24-min assay. HD video was recorded at 15 frames/second.

#### 2.3.5. Mortality and Morphology Response

Embryos were exposed statically and assessed at 24 hpf for four developmental toxicity endpoints (mortality, developmental progression, spontaneous movement, and notochord distortion), and again at 120 hpf for 18 developmental endpoints [60] by the Zebrafish Acquisition and Analysis Program (ZAAP) custom program, with evaluation conducted by evaluators from Sinnhuber Aquatic Research Laboratory.

### 2.4. Statistical Analysis

#### 2.4.1. Zebrafish Exposure to Pigment Extracts

Statistical analysis followed methods in Truong et al. [60]. Binary data from zebrafish assessments were compared using the Fisher’s exact test. This statistically compared the mortality of fish embryos exposed to pigments versus a baseline five percent mortality rate of control embryos, using the *proc freq* function in SAS 9.4.

#### 2.4.2. Zebrafish Exposure to Solid Pigments

Zebrafish exposure to solid pigments analysis followed analysis in Troung et al. [63]. Embryo Photomotor Response (EPR) behavior was analyzed by comparing the background, excitatory, and refractory intervals to the negative control (0 μg/mL of compound) activity using a combination of percent change and a Kolmogorov–Smirnov test (Bonferroni-corrected *p*-value threshold). Dead or deformed fish were excluded from behavioral datasets.

Larval photomotor response movement data from the behavior chamber were integrated into 6 s bins, and the area under the curve was compared to control movement via *t*-test for each exposure concentration. LPR was considered valid when percent change in area under the curve was greater than or equal to 40% above the control group and statistical significance (at *p* < 0.05) was met. Dead or deformed fish were excluded from behavioral datasets.

Statistical analysis of mortality and morphology endpoints was performed in R [64], based on binary indices for each endpoint (*n* = 32). A significance threshold was computed for each chemical and endpoint combination in comparison to the control incidence rate, and Fisher’s exact test was used to compare treatment groups to control groups to account for low category counts. Control data were used to check for confounding plate, well, and chemical effects. Slight differences in chemical effects lead to multiple comparisons used to control the family wise error rate.

Concentration response modeling based on mortality and morphology data was carried out on mortality data at 24 hpf and at 120 hpf for tested compounds showing significant responses compared to the control. R was used to fit a Hill model to the average of all individuals at each exposure concentration following methods in Truong et al. [63], using the four parameters of lower limit, upper limit, the median effective concentration (EC50) curve inflection point, and the “Hill” slope. Curves were fit with the *drm()* function in *drc* package in R, using least squares estimation. The strength of each curve was assessed for goodness of fit using Normalized Root Mean Square Error and Akaike Information Criterion.

## 3. Results and Discussion

### 3.1. Solvent-Extracted Pigment and Liquid Culture Testing

#### 3.1.1. Pigment from Solvent Extraction (No Purification)

**Figure 2:**
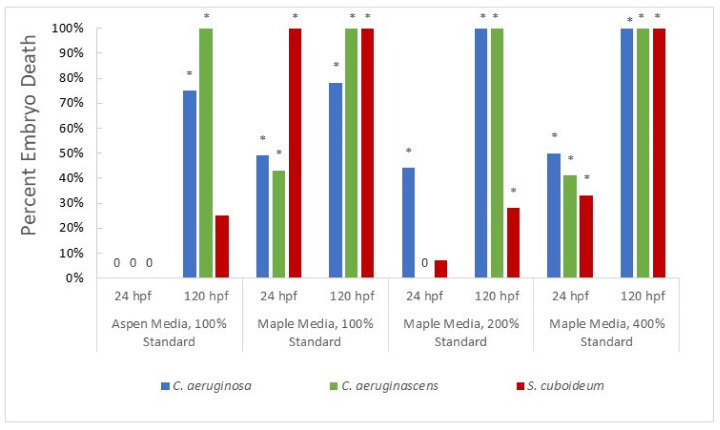
Percent Embryo Death After Exposure to Dichloromethane Extracted Pigments from Fungi at 24 and 120 hpf. Asterisks denote significant difference from control, with all significant values having a *p*-value of <0.0001 as determined by Fisher’s exact test. Zeros indicate there was no fish mortality in that condition. DCM-extracted pigment is shown to result in significantly more mortality than control across a number of test conditions, with higher embryo death rates after longer exposure. Aspen media resulted in less mortality than maple media, and higher concentrations of maple media resulted in more deaths for pigments from most fungal species.

Dichloromethane-extracted pigment resulted in significantly more mortality than the control across a number of test conditions (Figure 2), including tested pigments, exposure concentrations, and fungal growth substrate. Mortality results were also reflected in the number of embryos with sublethal effects, presented in Figure S1. Deformations seen included pericardial edema, yolk sac edema, reduced total length, and axis problems, with deflated swim bladder and bent tail/trunk seen in 400× standard concentration.

**Figure 3:**
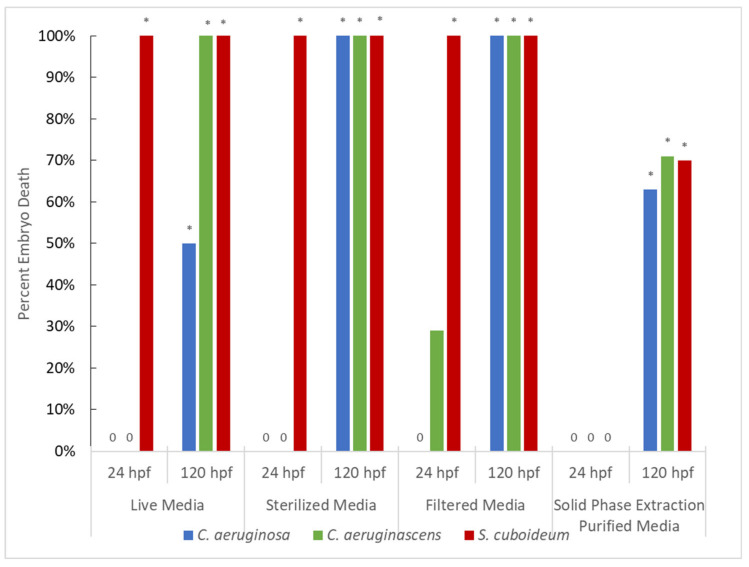
Percent Embryo Death After Exposure to Pigments from Fungi Grown in Liquid Media at 24 and 120 hpf. Asterisks denote significant differences from control at the α = 0.05 level, with all significant values having a *p*-value of <0.0001, apart from live media *C. aeruginosa* at 120 hpf which had a *p*-value of 0.0028. Zeros indicate there was no embryo mortality in that condition.

The toxicity of pigments varied based on if fungi were grown in wood chips amended with either maple or aspen. Pigments from fungi grown in maple media resulted in significantly more deaths at 24 hpf than the control for all fungal species, most notably in the case of *S. cuboideum* where exposure resulted in 100% mortality. In contrast, no death or deformity was seen in extracts from any fungi grown on aspen at 24 hpf, though by 120 hpf both species of *Chlorociboria* showed significant deaths and high levels of deformity (Figure 2 and Figure 3). The two wood varieties have differing extractive profiles [65], with maple containing bioactive compounds such as resorcinol [66], which likely accounts for these differences. *Chlorociboria aeruginascens* resulted in complete mortality by 120 hpf in all tested conditions, with *C*. *aeruginosa* showing similar toxicity. Both species also showed high rates of sublethal effects, with deformations seen in all or nearly all embryos by 120 hpf (Supplemental Figure S1). This suggests that other bioactive secondary metabolites are likely produced by *Chlorociboria* species in amended plate cultures. Finally, *S. cuboideum* extract showed total mortality by 24 h at 100% concentration; however, it did not have significantly different mortality than the control at the same time under 200% exposure (*p* = 1.000) and did not show complete mortality by 120 hpf. This suggests that there may be variation in embryo response, leading to inconsistency in the lethality of pigment extract, though the eventual significance of embryo mortality indicates overall toxicity. 

#### 3.1.2. Pigment from Liquid Culture

Pigments from liquid cultures were associated with high rates of mortality, with all pigments across test conditions resulting in mortality significantly different than the control by 120 hpf (Figure 2). *Scytalidium cuboideum* liquid culture pigment was especially lethal, with all embryos dead at 24 h in every test condition except SPE purified pigment (Figure 2). This was a contrast compared to DCM-extracted pigments, which did not show the same consistent toxicity. The differences in toxicity seen between the liquid cultures and the solid cultures are likely explained by variation in differential metabolite production. Variation in growth conditions is known to alter the secondary metabolite production of fungi generally [67,68,69], and variation in metabolite production in spalting fungi specifically [70]. Increased co-production of bioactive compounds in the liquid cultures compared to that in the wood chip plates is likely, especially as pigmented metabolite production is often seen earlier in the lab in liquid compared to solid cultures. In addition to this effect, differences between liquid cultures and DCM extracted pigment were likely influenced by the differences in pigment concentration, the presence of growth media in liquid cultures, and the likelihood that DCM extract contains fewer products of fungal metabolism due to its polarity limiting transfer of compounds.

At 24 hpf there were differences between fungal species, with *Chlorociboria* species showing no significant toxicity in live, sterilized, or filtered media. However, *C. aeruginascens* showed relatively high sublethal effects at 24 hpf despite the lack of significant mortality (Figure S2). This variation in the production of secondary metabolites in addition to target pigment was also observed in DCM-extracted cultures, while both *Chlorociboria* spp. showed high levels of toxicity in DCM- and liquid media solutions. This suggests that other fungal metabolites may have been responsible for the effects seen, especially as toxicity was also lower in SPE-purified samples compared to liquid media and there appeared to be variation in toxicity between species. *Chlorociboria aeruginosa* showed lower percent mortality than *C. aeruginascens* in multiple tests. Notably, in live media testing at 120 hpf, *C. aeruginosa* showed only half the mortality of *C. aeruginascens*, and in sterilized media at 24 hpf it showed no toxicity while *C. aeruginascens* had 100% deformities. Variation in pigment production between the two species has been seen in other studies [70,71], including variation in the production of a yellow pigment in addition to differential production of xylindein [54].

SPE purification resulted in no mortality seen at 24 hpf, though by 120 hpf there was significant mortality for all tested pigments (Figure 2) and high levels of total sublethal effects. Deformations observed included pericardial edema, yolk sac edema, trunk, axis, and craniofacial malformations.

### 3.2. Solid Pigments and Behavior Response Testing

#### 3.2.1. Embryo Photomotor Response 

The red pigment crystal dramada produced by *S. cuboideum* was the only compound shown to have significant bioactivity. Exposure to concentrations of 10.7 and 23.2 µM was associated with hyperactive tail bending in the excitatory phase (*p* = 0.043 and 0.022, respectively), and in the case of 23.2 µM also in the baseline phase (*p* = 0.007). In contrast, at 50 µM an absence of any activity was observed in both the excitatory and baseline phases, with *p* values of 0.024 and 0.022, respectively. 

#### 3.2.2. Larval Photomotor Response

Dramada was also the only tested compound that showed any significant effect on larval photomotor response, though all tested compounds were modestly bioactive. Many were associated with effects during the light phase of testing, with the exception of one concentration of the red pigment dramada. Aniline was shown to have a significant hyper effect on photomotor response behaviors in the light interval at 2.32 µM (*n* = 27, *p* < 0.01), at 5 µM (*n* = 30, *p* < 0.01), at 10.7 µM (*n* = 26, *p* < 0.01), and at 23.2 µM (*n* = 27, *p* < 0.01). Xylindein from *C. aeruginosa* was associated with a hyperactive response on LPR in light at ~5 mM or 2.8 mg/mL (*n* = 15, *p* < 0.01), and a hypoactive response at ~10.7 mM or 5.7 mg/mL (*n* = 14, *p* < 0.01). Finally, dramada from *S. cuboideum* at 2.32 mM showed a hypoactive effect in both dark (*n* = 26, *p* < 0.01) and light (*n* = 26, *p* < 0.01) conditions, and a hypoactive effect in light at 5 mM (*n* = 23, *p* < 0.01) and 10 mM (*n* = 23, *p* < 0.01). Dramada also did not have enough animals remaining at 120 hpf to allow for analysis, unlike xylindein and aniline which showed no significant effects at the highest concentration. These abnormalities seen in dramada and impure xylindein from *C. aeruginosa* were modest in comparison to other known compounds that have a major effect on LPR in the lighted phase [42]. In addition, the lack of association with differences in the dark phase is unusual, suggesting that the effect may be at the level of detection and spurious. The control compound used for comparison, aniline, showed similar moderate levels of abnormalities. 

#### 3.2.3. Mortality and Morphology

**Figure 4:**
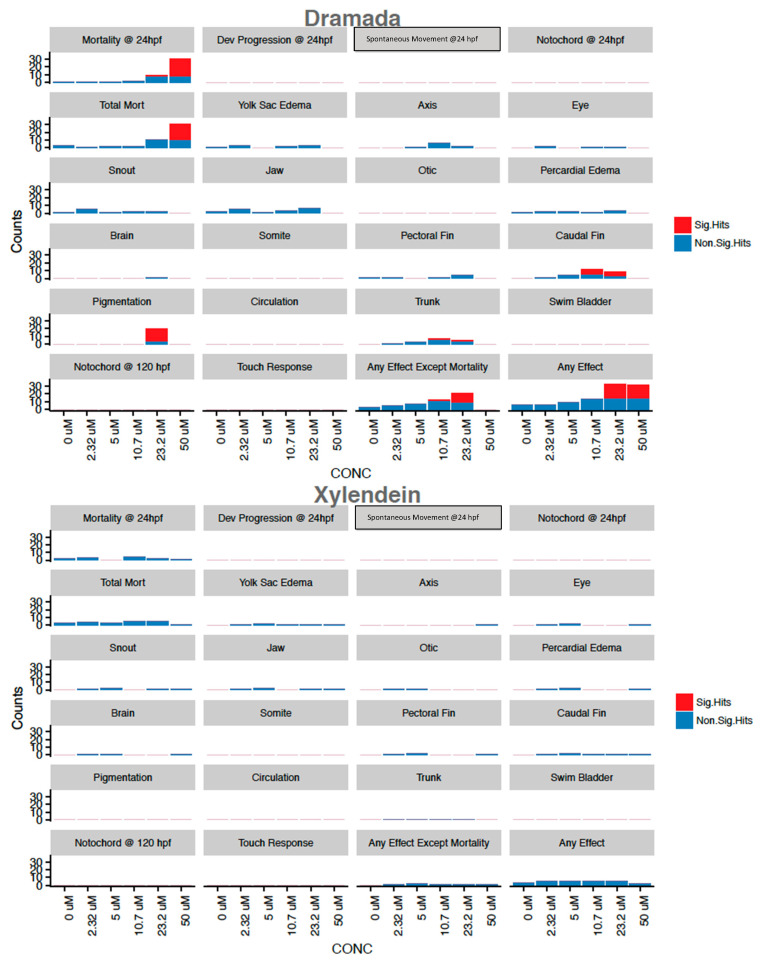
Mortality and Morphology Endpoint Counts for Dramada and Xylindein. Presence/absence data across 32 replicates with points above and at threshold for binomial significance in red. Concentration for xylindein is approximate due to lack of pure compound for testing. Control incidence of all morphological and touch response endpoints below 20% cutoff for biological validity.

Dramada was the only tested compound that exhibited significant morbidity in tested endpoints, with 50 µM concentrations associated with near 100% mortality by 24 hpf (Figure 4). Deformations found to be significantly associated with exposure included caudal fin deformity at 10.7 and 23.2 µM, abnormal pigmentation at 23.2 µM, and modest body length shortening at 10.7 and 23.2 µM. Concentration response modeling for dramada showed that the LC_50_ for mortality at 24 hpf was estimated to be 38.2 µM (±1.4), and mortality (LD_50_) at 120 hpf was estimated to be 25.5 µM (±3.4). This higher bioactivity is not surprising, as many other naphthoquinones from various sources are known to have bioactive properties [72], and the red pigment has been previously described as having modest bioactivity against Gram-negative bacteria and fungi [16]. Other red naphthoquinonic pigments extracted from filamentous fungi have shown cytotoxicity against cell lines [73,74], and one of these compounds, erythreostominone, has also been shown to induce malformations and impair locomotor activity [75,76].

In contrast, xylindein and aniline showed non-significant toxicity responses without a clear dose-response relationship (Figure 4). Aniline is associated with known toxic and potential mutagenic effects [77], and has been shown to have developmental and sublethal effects in zebrafish [78] with reported LD_50_ values at 96 h of 618 (±43.0) µmol/L [79], higher than the concentrations tested here. 

Based on the ratio of LD_50_ value to body mass, it would take 510.4 kg (2040 M at 250.20 g/mol) of dramada for an 80 kg (~176 lbs) human to experience equivalent exposure to the 25.5 µM experienced by a 1 mg embryo – while also being fully immersed in pigment. For reference, the LD_50_ of table salt taken orally is 3 g/kg for a rat [80], equivalent to 240 g for an 80 kg human. Previous work on the toxicity of the red pigment when isolated from an actinomycete showed it to be non-toxic at 600 mg/kg administered intraperitoneally to mice [16]. These factors suggest that while dramada may present some toxicity, it may not present a human health risk except at extreme quantities.

The applications developed based on dramada from *Scytalidium cuboideum* have focused on its use as a coloring agent for textiles and bamboo [20,21,58], using a standard concentration of extracted pigment. Estimates of the pigment concentration in this “standard” pigment solution range from 0.73 to 2.4 mM [55], making the standard used much higher than the calculated LD_50_ of 25.5 µM (±3.4) at 120 hpf. This suggests that solutions used in current methods likely contain quantities of pigment that may pose a risk for developing embryos, if the pigments were transferred to water to allow for exposure.

However, while dramada has demonstrated a toxic effect on zebrafish, this result does not necessarily prevent its use in industry. Many dyes currently in use in the textile industry have been shown to have toxic effects [81], and the development of natural dyes to replace toxic synthetics has been a focus of research [82]. Additionally, as the processes currently used for dyeing with dramada do not produce wastewater [59,83,84], there is a limited likelihood of effluent reaching watersheds and impacting fish development. In addition, dramada has limited solubility in water and strongly binds to materials such as textiles, wood, and glass, making transfer and high levels of exposure unlikely. While any toxicity suggests that caution should be taken in regard to the use of the pigment for applications where consistent exposure to humans is expected, further testing of prepared textiles and other materials should be carried out to determine bioavailability and the likelihood of exposure.

While the red pigment produced by *S. cuboideum* demonstrates some toxicity, the green pigment produced by *Chlorociboria* spp. in solid form has limited bioactivity. However, pigmented fungal media solutions and, in some cases, extraction using DCM were associated with significant mortality. This suggests the co-production of other toxic secondary metabolites, while xylindein itself may be non-toxic. Purer *Chlorociboria* extract has also been shown to have improved semiconductive capabilities [56], further supporting the need for improved methods of xylindein purification. As xylindein is under investigation for use in solar cells, an industry associated with toxic byproducts and materials [85,86,87], the adoption of xylindein as an alternative could improve the environmental friendliness of energy generation in addition to its sustainability. Future work should focus on the identification and removal of possible mycotoxins and the use of purified pigments. This research supports previous research into the use of these pigments for a variety of applications, allowing for future industrial adoption. The use of these sustainably sourced pigments with limited toxicity has the potential to improve sustainability and replace toxic byproducts across multiple industries.

## 4. Conclusions

Pigments from spalting fungi show varying levels of toxicity to zebrafish embryos across species and growth/extraction methodology. The red pigment dramada from *S. cuboideum* was the only solidified and pure pigment associated with significant toxicity in zebrafish, though with a relatively high LD_50_ value, making it unlikely to affect humans. Solidified and washed xylindein did not demonstrate toxicity. However, significant mortality was associated with impure solutions of fungal metabolites containing pigments from all tested species. This suggests the co-production of mycotoxins or toxicity related to media components. The development of improved purification methodologies, especially for xylindein from *Chlorociboria* species, is therefore of paramount importance for future industrial adoption. However, the low levels of toxicity seen in the solidified xylindein are sufficient to suggest that future technologies are likely to be both sustainable and environmentally safe. The adoption of these sustainably produced pigments has the potential to replace conventional technologies currently associated with toxicity, allowing for a greener future.

## Data Availability

Not applicable.

## Supplementary Materials

The following are available online at https://www.mdpi.com/2309-608X/7/2/155/s1, Figure S1: Percent of Embryos Showing Sublethal Effects After Exposure to DCM-Extracted Pigments from Fungi at 24 and 120 hpf, Figure S2: Percent of Embryos Showing Sublethal Effects After Exposure to Pigments from Fungi Grown in Liquid Media at 24 and 120 hpf.
